# Prescription sequence symmetry analysis of voriconazole-associated hypomagnesemia in pediatric patients with hematologic diseases

**DOI:** 10.3389/fphar.2026.1948603

**Published:** 2026-09-09

**Authors:** Ying Li, Han Xie, Linbo Tan

**Affiliations:** Department of Pharmacy, Children’s Hospital of Chongqing Medical University, National Clinical Research Center for Children and Adolescents' Health and Diseases, Ministry of Education Key Laboratory of Child Development and Disorders, Intelligent Application of Big Data in Pediatrics Engineering Research Center of Chongqing Education Commission of China, Chongqing, China

**Keywords:** hematologic diseases, hypomagnesemia, pediatrics, pharmacovigilance, prescription sequence symmetry analysis, therapeutic drug monitoring, voriconazole

## Abstract

**Objective:**

To evaluate the temporal association between voriconazole and hypomagnesemia in pediatric patients with hematologic diseases using prescription sequence symmetry analysis (PSSA) and to assess the utility of hospital information system (HIS)-based active drug safety surveillance.

**Methods:**

Inpatient data were extracted from the Children’s Hospital of Chongqing Medical University from November 2013 to December 2024. The first year served as a data integrity verification window and the subsequent approximately 10-year period as the formal analysis phase. Patients with hematologic diseases were identified using ICD-10 codes C81–C96, D45–D47, and D60–D61. Each patient contributed the first voriconazole prescription and first hypomagnesemia event (Mg^2+^ < 0.6 mmol/L). The primary analysis used a 365-day washout period, 90-day exposure window, and 7-day blackout period. Adjusted sequence ratios (aSRs) with 95% confidence intervals (CIs) were estimated, with 10 parameter sensitivity analyses, monitoring-intensity correction, four control analyses, and stratified analyses by concomitant medications.

**Results:**

Among 536 eligible patients, 364 (67.9%) had serum magnesium measurements. The post-exposure sequence comprised 58 patients and the reverse sequence eight patients, yielding an aSR of 5.76 (95% CI: 2.75–12.06). Median time to hypomagnesemia after voriconazole initiation was 28 days (IQR: 20–43), and 32/58 events (55.2%) occurred within 30 days. Exploratory dose-response analysis showed no association between weight-based voriconazole dose and hypomagnesemia occurrence (P = 0.437). All parameter sensitivity analyses produced aSRs >3.9; after correction for monitoring intensity, the aSR was 3.33 (95% CI: 1.59–6.98); in a sensitivity grid, the association remained statistically significant for monitoring-intensity ratios up to 2.75. All four control analyses agreed with their prespecified expectations. Associations remained significant in the PPI and aminoglycoside strata, whereas the loop-diuretic non-user stratum showed the same direction but was not statistically significant.

**Conclusion:**

Voriconazole initiation was temporally associated with subsequent hypomagnesemia in pediatric patients with hematologic diseases. PSSA may complement spontaneous reporting systems for detecting laboratory-defined safety signals. Weekly serum magnesium monitoring should be considered during the first 30 days of voriconazole therapy. These findings support a temporal association but do not establish causality.

## 1 Introduction

Voriconazole, a second-generation triazole antifungal, is widely used for the prevention and treatment of invasive fungal infections in patients with hematologic diseases (Patterson et al., 2016). The FDA prescribing information reports hypomagnesemia as an adverse reaction in pediatric patients (U.S. Food and Drug Administration, 2025). However, this adverse event may be underestimated in real-world clinical practice for three reasons. First, electrolyte panels in many Chinese tertiary hospitals include only potassium, sodium, and chloride, whereas serum magnesium is not routinely measured, potentially resulting in under-detection of hypomagnesemia. Second, the clinical manifestations of hypomagnesemia, including muscle weakness, tremor, and QTc prolongation, may overlap with manifestations of the underlying disease and treatment-related complications, making attribution to a single cause difficult. Third, proton pump inhibitors (PPIs), loop diuretics, and aminoglycosides—all known to cause hypomagnesemia (Gommers et al., 2022; Rosner et al., 2023; Mingeot-Leclercq and Tulkens, 1999)—may be co-administered during intensive treatment of hematologic diseases. Consequently, detected hypomagnesemia may be attributed to these concomitant medications, while the possible contribution of voriconazole may receive less attention.

Spontaneous reporting systems such as the FDA Adverse Event Reporting System (FAERS) exhibit a monitoring blind spot for laboratory abnormalities that do not cause subjective discomfort—a surveillance system that “hears gunshots but not whispers.” Hypomagnesemia is precisely the signal that murmurs persistently in the noisy hematology ward yet is seldom heard. Xiao et al. (2024) and Li et al. (2025) both failed to detect a voriconazole–hypomagnesemia signal in their FAERS analyses.

Prescription sequence symmetry analysis (PSSA), first proposed by Hallas in 1996 (Hallas, 1996), is a self-controlled pharmacoepidemiological design that evaluates the temporal association between a drug and an event by comparing the number of patients with the sequence “prescription→event” (npost) versus “event→prescription” (npre). The self-controlled rationale is that each patient serves as their own control: time-invariant patient characteristics (e.g., genetic background, chronic comorbidities, socioeconomic status) are inherently balanced because the same individual contributes to both sequence directions. The key assumptions are that the index exposure and outcome are recorded with reasonable temporal precision, that the outcome does not alter the probability of the exposure being prescribed (no reverse causation), and that exposure initiation is not strongly influenced by the outcome itself (King et al., 2020; Pratt et al., 2015). PSSA relies entirely on existing prescription and laboratory data, making it well suited to hospital information system-based surveillance. Compared with other self-controlled designs, PSSA shares the within-person control principle of case-crossover and self-controlled case series analyses but differs in its reliance on aggregate sequence counts rather than individual-level event rate modelling; it is computationally simple, requires only two event dates per patient, and is particularly efficient for screening analyses, although it is less flexible than self-controlled case series for modelling time-varying exposures and is susceptible to prescribing time trends, which are addressed through the null-effect sequence ratio (King et al., 2020; Gault et al., 2017). A five-country validation study by Pratt et al. confirmed the consistency of PSSA estimates across different health settings (Pratt et al., 2015), and a validation study by Wahab et al. reported a sensitivity of 61% and specificity of 93% against randomized controlled trials as the gold standard (Wahab et al., 2013). To date, PSSA has not been applied to evaluate antifungal drug–electrolyte disorder associations.

To the best of our knowledge, the present study is the first to apply PSSA to the domain of antifungal drugs and hypomagnesemia, with three objectives: to explore the temporal association between voriconazole and pediatric hypomagnesemia; to advance PSSA practice standards in China through ICD-10-based precision screening, sensitivity analysis grids, and Pratt control validation; and to provide a reproducible technical framework for HIS-based active drug safety surveillance. In addition, we conducted an exploratory analysis of the relationship between weight-based voriconazole dose and the occurrence of hypomagnesemia.

## 2 Materials and methods

### 2.1 Study design and data sources

This retrospective PSSA study used data from the Hospital Information System (HIS) of the Children’s Hospital of Chongqing Medical University. Inpatient data were extracted for pediatric patients with voriconazole administration records between 13 November 2013 and 4 December 2024, including medication administration records, laboratory test results, diagnostic information, and visit records. Records belonging to the same patient across multiple visits, prescriptions, and laboratory tests were linked through internal database identifiers.

The first year (13 November 2013 to 12 November 2014) was designated as the data integrity verification window, and the subsequent period (13 November 2014 to 4 December 2024) served as the formal analysis phase. The verification window was used to assess whether apparently first voriconazole prescriptions and hypomagnesemia events in the formal analysis phase could reflect left-truncated prior histories. Retrospective review showed that the first voriconazole prescription among the 536 patients and all qualifying first hypomagnesemia events used to construct PSSA sequences occurred after the verification window; the earliest qualifying record occurred in 2017. Each analyzed sequence therefore had more than 3 years of observable baseline before the first qualifying prescription or event, satisfying the 365-day washout requirement, and no sequence was excluded because of an insufficient observable baseline.

**FIGURE 1:**
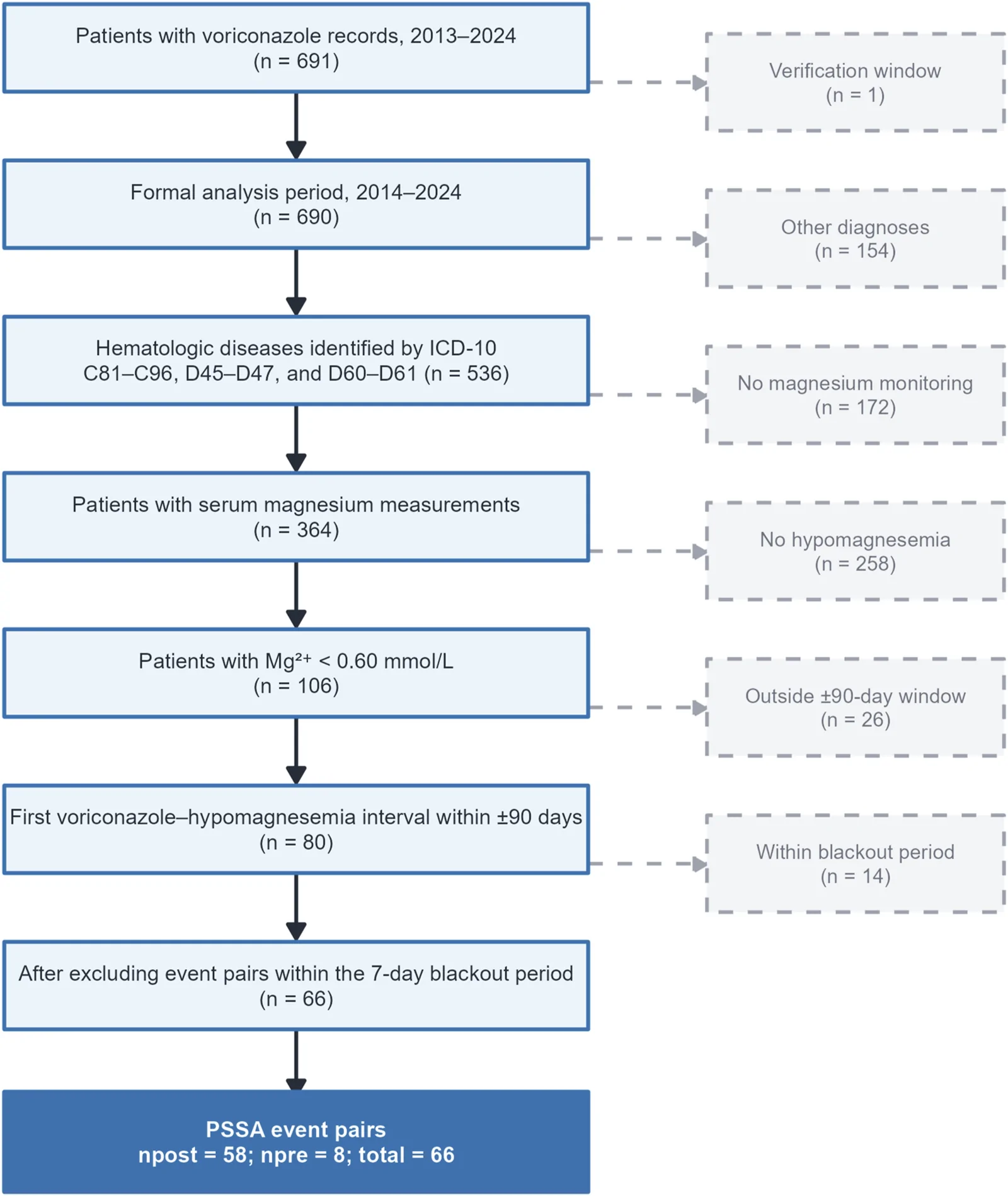
Participant selection and construction of event pairs for prescription sequence symmetry analysis. The verification window covered 13 November 2013 to 12 November 2014, and the formal analysis period covered 13 November 2014 to 4 December 2024. PSSA, prescription sequence symmetry analysis; Mg, magnesium; ICD-10, International Classification of Diseases, 10th Revision.

Patients with hematologic diseases were identified using a prespecified ICD-10 coding strategy: C81–C96 (malignant neoplasms of lymphoid, hematopoietic, and related tissue), D45–D47 (myelodysplastic, myeloproliferative, and related disorders), and D60–D61 (acquired pure red cell aplasia and other aplastic anemias). Compared with keyword-based matching, the use of prespecified ICD-10 codes improves the reproducibility of population identification across investigators and institutional information systems (Figure 1; Supplementary Table S1).

### 2.2 Unit of analysis

The patient was used as the unit of analysis, with each patient contributing only one event pair (first voriconazole prescription and first hypomagnesemia event). Among the study population, 312 patients (58.2%) experienced at least two hospitalizations during the study period, corresponding to a total of 1,882 hospitalization records for the 536 patients. Using individual hospitalizations as the unit of analysis would treat multiple hospitalizations of the same patient as independent events, violating the independence assumption of PSSA. All records for a given patient were linked through a unique internal patient identifier across hospitalizations, including those separated by several years. Recurrent voriconazole exposure was addressed by restricting the analysis to the first qualifying prescription per patient, and recurrent hypomagnesemia was addressed by restricting the analysis to the first qualifying event per patient.

### 2.3 Index drug and outcome event

The index drug was voriconazole, including both oral and intravenous formulations. The outcome event was hypomagnesemia, with Mg^2+^ < 0.60 mmol/L used as the primary threshold. This threshold falls within the mild-to-moderate range of hypomagnesemia in pediatric oncology patients (Kaplinsky and Alon, 2013) and was selected to capture clinically relevant events while maintaining adequate event counts; alternative thresholds (<0.55, <0.65, <0.70 mmol/L) were examined in sensitivity analyses. Although this exploratory analysis was considered in relation to the Bradford Hill biological-gradient criterion (Hill, 1965), it varied outcome severity rather than voriconazole dose and was therefore not interpreted as a formal dose-response analysis; a separate exploratory analysis of weight-based voriconazole dose is described in Section 2.9. Patients were eligible only if no qualifying hypomagnesemia event had occurred within 365 days before the index voriconazole prescription, ensuring that the observed event represented an incident outcome rather than persistence or recurrence of a prior episode.

### 2.4 Time window parameters

Washout period (365 days): patients with a prior qualifying voriconazole exposure or outcome event during the preceding 365 days would be excluded to support the identification of incident exposure and outcome events. Retrospective baseline verification identified no such prior records. Sensitivity analyses using washout periods of 180 and 540 days did not alter the event-pair counts.

Exposure window (90 days): defined the maximum acceptable interval between prescription and event. This was informed by prior PPI-PSSA research (PPI-induced hypomagnesemia involves TRPM6 channel inhibition, a known mechanism for drug-induced hypomagnesemia (Gommers et al., 2022)) and was validated at 30, 60, and 180 days in sensitivity analyses. A 90-day window was chosen *a priori*, consistent with the exposure windows used in previous PSSA validation studies (Pratt et al., 2015; Wahab et al., 2013).

Blackout period (7 days): excluded event pairs occurring within a very short interval around the prescription date that might reflect clinical workflow, including delays between medical ordering, execution, specimen collection, and result reporting, rather than an interpretable temporal sequence. Sensitivity analyses using blackout periods of 0 and 14 days were performed.

### 2.5 Statistical analysis

The PSSA procedure comprised four steps. First, the crude sequence ratio was calculated as cSR = npost/npre. Second, to estimate the null-effect sequence ratio (SRnull), outcome dates were randomly permuted among patients with both a first voriconazole prescription and a first qualifying outcome event 2,000 times using a fixed random seed of 42; the 7-day blackout period and 90-day exposure window were reapplied in each iteration, and SRnull was calculated as the mean of the permuted post-exposure-to-reverse sequence ratios. The permutation approach accounts for secular prescribing trends by preserving the empirical distribution of event dates relative to prescription dates under the null hypothesis of no drug-event association; because prescription volumes increased markedly during the study period (annual first-time voriconazole users: 1, 2, 5, 16, 44, 149, 254, and 65 in 2017–2024, respectively), the null-effect sequence ratio is expected to exceed 1, and the permutation estimate directly captures this imbalance. The fixed random seed of 42 ensures reproducibility. Third, the adjusted sequence ratio was calculated as aSR = cSR/SRnull. Fourth, 95% CIs were estimated using a Wald approximation on the logarithmic scale, with the standard error calculated as √(1/npost +1/npre). A signal was considered positive when aSR >1 and the lower limit of the 95% CI exceeded 1.0 (Hendrix et al., 2024). Analyses were performed using Python 3.11 (pandas 2.0, numpy 1.24, scipy 1.10). The analysis code is provided in the Supplementary Material.

### 2.6 Sensitivity analyses

In addition to the main analysis, 10 parameter sensitivity analyses covering four parameter dimensions were designed, together with a separate monitoring intensity correction: Mg^2+^ thresholds (0.55/0.60/0.65/0.70 mmol/L), washout periods (180/365/540 days), exposure windows (30/60/90/180 days), and blackout periods (0/7/14 days). All sensitivity analyses employed the identical analytical workflow, varying only the target parameter while holding all other parameters at their main-analysis settings, ensuring that differences in results were attributable solely to parameter variation.

### 2.7 Control analyses

The four control outcomes and their thresholds were prespecified in the study protocol before the main analysis was conducted. Following the Pratt et al. five-country validation framework (Pratt et al., 2015) and the Vouri et al. negative control strategy (Vouri et al., 2021), four controls were established within the same dataset: (1) true-negative: hyponatremia (Na^+^ < 130 mmol/L), for which voriconazole has no pharmacological pathway affecting sodium metabolism; (2) weak-negative: elevated total bilirubin (TBil >34 μmol/L), an occasionally reported adverse event for voriconazole but with a causal strength far lower than ALT elevation; (3) weak-positive: hypokalemia (K^+^ < 3.0 mmol/L), a known adverse reaction explicitly listed in the FDA prescribing information (U.S. Food and Drug Administration, 2025); and (4) positive: ALT elevation (>80 U/L, well above the upper limit of the pediatric reference range) (Schwimmer et al., 2010), the most thoroughly documented adverse reaction of voriconazole, with an overall reporting rate exceeding 10%.

### 2.8 Concomitant medication assessment

Stratified PSSA analyses were performed according to exposure to PPIs (Gommers et al., 2022), loop diuretics (Rosner et al., 2023), and aminoglycosides (Mingeot-Leclercq and Tulkens, 1999) within ±30 days of voriconazole initiation. Persistence of the association in the non-user stratum was interpreted as evidence that the observed association was not entirely explained by the concomitant medication; however, residual and time-varying confounding could not be excluded. Platinum-based agents (4 exposed patients) and amphotericin B (51 exposed patients) were not subjected to stratified analysis because the exposure numbers were considered insufficient to support stable stratum-specific estimates.

### 2.9 Exploratory dose-response analysis

An exploratory analysis of the relationship between weight-based voriconazole dose and hypomagnesemia was prespecified as a secondary objective. Daily voriconazole dose was calculated from medication administration records as the product of the single dose and dosing frequency (mg/day) and standardized by body weight recorded at the index hospitalization (mg/kg/day); the maximum daily dose within the first 14 days of the index voriconazole course was used to characterize exposure intensity. Patients were classified into tertiles of weight-based dose, and the 90-day incidence of hypomagnesemia (Mg^2+^ < 0.60 mmol/L) was compared across tertiles using the Cochran–Armitage trend test; the high-versus low-tertile risk ratio (RR) with a chi-square test was reported as a complementary comparison. Weight-based dose and the number of voriconazole administrations (a proxy for treatment duration) were additionally compared between patients with and without hypomagnesemia using the Mann–Whitney U test.

The reporting of this study follows the Reporting of studies Conducted using Observational Routinely-collected health Data (RECORD) statement; the completed checklist is provided in the Supplementary Material.

## 3 Results

### 3.1 Baseline characteristics

During the formal analysis period, 536 pediatric patients with hematologic diseases received voriconazole, of whom 364 (67.9%) had serum magnesium measurements and were included in the PSSA analysis. Among these 364 patients, 230 (63.2%) were male and 134 (36.8%) were female. The median age was 6.1 years (IQR 3.3–9.9, range 0.2–17.1). The principal visiting departments—Day Care Internal Medicine, Hematology-Oncology Ward 1, and Hematology-Oncology Ward 2—accounted for over 85% of all visit records. A total of 7,955 serum magnesium measurements were performed during the study period, with a median of 20 measurements per patient (IQR 7–29) and a median serum magnesium value of 0.79 mmol/L (Table 1).

**TABLE 1:** Baseline characteristics of the study population.

Characteristic	Value
Study period	2014-11-13 to 2024-12-04
Patients with hematologic diseases receiving VRC, n	536
With serum Mg monitoring, n (%)	364 (67.9)
Sex (among monitored), male/female, n (%)	230 (63.2)/134 (36.8)
ICU admission, n (%)	8 (2.2)
Renal disease diagnosis, n (%)	0 (0)
Age, years, median (IQR, range)	6.1 (3.3–9.9, 0.2–17.1)
Total serum Mg measurements	7,955
Mg measurements per patient, median (IQR)	20 (7–29)
Median serum Mg, mmol/L	0.79
Ethnicity: Chinese, n (%)	536 (100)
Concomitant medication within ±30 days of VRC initiation	n (%)
PPI	166 (45.6)
Loop diuretic	124 (34.1)
Aminoglycoside	186 (51.1)

VRC, voriconazole; Mg, magnesium. Concomitant medication defined as any administration within ±30 days of the first voriconazole prescription. ICU, intensive care unit; PPI, proton pump inhibitor.

### 3.2 Main analysis

**FIGURE 2:**
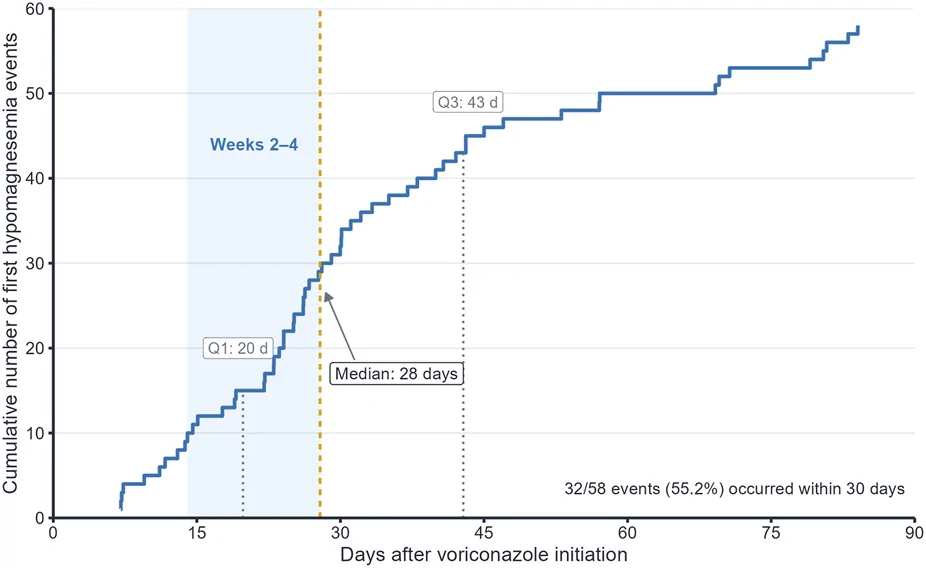
Cumulative distribution of time to first hypomagnesemia after voriconazole initiation. The analysis included 58 events in the voriconazole-to-hypomagnesemia direction. The median time to onset was 28 days (interquartile range 20–43 days), and 32 of 58 events (55.2%) occurred within 30 days. The shaded area denotes weeks 2–4 after voriconazole initiation; dashed vertical lines indicate the first and third quartiles (Q1 and Q3).

Using Mg^2+^ < 0.60 mmol/L as the primary analysis threshold, 58 patients had the post-exposure sequence (voriconazole→hypomagnesemia) and 8 had the reverse sequence (hypomagnesemia→voriconazole), yielding a cSR of 7.250, an SRnull of 1.259, and an aSR of 5.76 (95% CI: 2.75–12.06) (Table 2). Among the 58 patients with the post-exposure sequence, the median interval from voriconazole initiation to hypomagnesemia was 28 days (IQR 20–43 days), and 32/58 events (55.2%) occurred within 30 days. Events were most concentrated during weeks 2–4 and accumulated more slowly after 60 days (Figure 2).

**TABLE 2:** Main analysis across hypomagnesemia thresholds.

Mg threshold (mmol/L)	n_post_	n_pre_	cSR	SR_null_	aSR	95% CI
<0.55	43	5	8.600	1.441	5.97	2.36–15.06
<0.60 (main)	58	8	7.250	1.259	5.76	2.75–12.06
<0.65	79	11	7.182	1.213	5.92	3.15–11.12
<0.70	113	21	5.381	1.152	4.67	2.93–7.44

cSR, crude sequence ratio; SR_null_, null-effect sequence ratio; aSR, adjusted sequence ratio; CI, confidence interval.

### 3.3 Analysis across hypomagnesemia thresholds

Across the 4 Mg^2+^ thresholds, all aSR estimates remained above 1: 4.67 at < 0.70 mmol/L, 5.92 at < 0.65 mmol/L, 5.76 at < 0.60 mmol/L, and 5.97 at < 0.55 mmol/L (Table 2). The estimates did not increase monotonically as the threshold decreased, and their 95% CIs overlapped substantially. These findings support the robustness of the association across alternative outcome definitions but do not establish a biological gradient or dose-response relationship.

### 3.4 Sensitivity analyses

**FIGURE 3:**
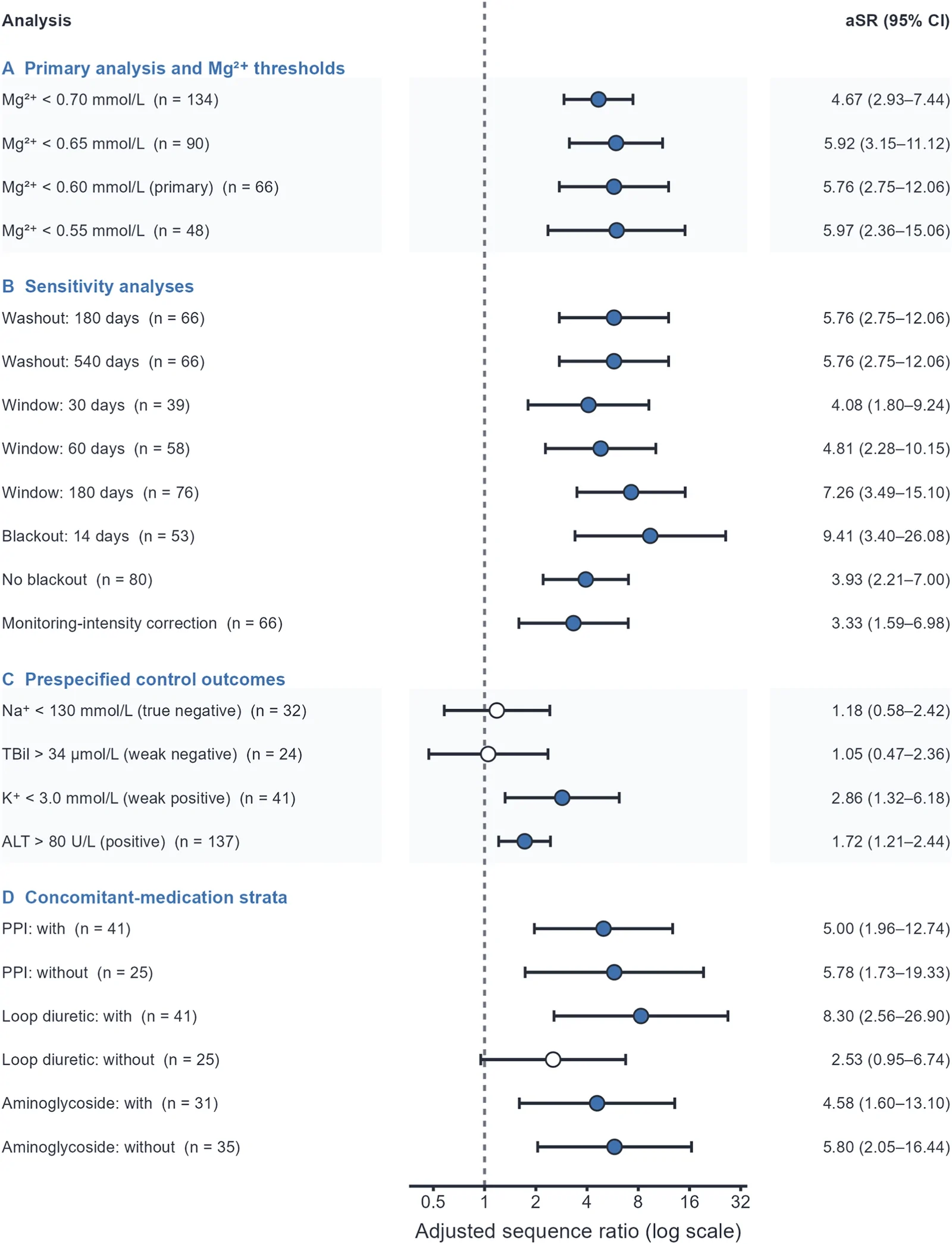
Adjusted sequence ratios across the primary, sensitivity, Pratt control, and concomitant-medication-stratified analyses. Points indicate adjusted sequence ratios and horizontal lines indicate 95% confidence intervals. The dashed vertical line indicates the null value of 1. Filled points denote estimates with 95% confidence intervals entirely above 1. aSR, adjusted sequence ratio; CI, confidence interval; PPI, proton pump inhibitor; TBil, total bilirubin; ALT, alanine aminotransferase.

The main analysis and all 10 parameter sensitivity analyses yielded aSR values >3.9, and the monitoring-intensity-corrected aSR was 3.33; the lower limits of all 95% CIs exceeded 1.0 (Table 3; Figure 3). Varying the washout period to 180 or 540 days produced results identical to the main analysis. Narrowing the exposure window to 30 days still yielded a statistically significant association (aSR = 4.08), whereas widening it to 180 days increased the aSR to 7.26, indicating that the estimated association was sensitive to the selected exposure window. Extending the blackout period to 14 days increased the aSR to 9.41. The proportional reduction in reverse-sequence events (50%) exceeded that in post-exposure-sequence events (16%), suggesting that reverse-sequence events were disproportionately concentrated near the prescription date and may partly reflect admission-related synchronous testing.

**TABLE 3:** Main analysis, parameter sensitivity analyses, and monitoring intensity correction.

Analysis	n_post_	n_pre_	aSR	95% CI
Main analysis	58	8	5.76	2.75–12.06
Mg < 0.55	43	5	5.97	2.36–15.06
Mg < 0.65	79	11	5.92	3.15–11.12
Mg < 0.70	113	21	4.67	2.93–7.44
Washout 180 d	58	8	5.76	2.75–12.06
Washout 540 d	58	8	5.76	2.75–12.06
Window 30 d	32	7	4.08	1.80–9.24
Window 60 d	50	8	4.81	2.28–10.15
Window 180 d	68	8	7.26	3.49–15.10
Blackout 14 d	49	4	9.41	3.40–26.08
No blackout	66	14	3.93	2.21–7.00
Monitoring adjustment	58	8	3.33	1.59–6.98

To assess the potential influence of monitoring bias, a *post hoc* analysis compared the number of serum magnesium measurements obtained during the 90 days after voriconazole initiation between the two sequence groups. The post-exposure-sequence group had a mean of 27.7 measurements per patient (median 25), compared with 16.0 measurements per patient (median 11.5) in the reverse-sequence group, corresponding to a monitoring intensity ratio of 1.73. Dividing the main-analysis aSR and its confidence limits by this ratio yielded an exploratory corrected estimate of 3.33 (95% CI: 1.59–6.98). Although this correction attenuated the estimate, the 95% CI remained above 1; however, the calculation should be interpreted as an exploratory bias analysis rather than a formally validated correction method (Table 3).

### 3.5 Control analyses

The results of the four Pratt control analyses are detailed in Table 4. The true-negative control (Na^+^ < 130 mmol/L; aSR = 1.18, 95% CI: 0.58–2.42) and the weak-negative control (TBil >34 μmol/L; aSR = 1.05, 95% CI: 0.47–2.36) both had CIs spanning 1.0, producing no false-positive signals. The positive control (ALT >80 U/L; aSR = 1.72, 95% CI: 1.21–2.44) and the weak-positive control (K^+^ < 3.0 mmol/L; aSR = 2.86, 95% CI: 1.32–6.18) both detected the expected positive signals.

**TABLE 4:** Prespecified control outcome analyses.

Control type	Analysis	n_post_	n_pre_	aSR	95% CI	Expected	Result
True negative	Na^+^ < 130	20	12	1.18	0.58–2.42	Negative	Negative
Weak negative	TBil >34	14	10	1.05	0.47–2.36	Negative	Negative
Weak positive	K^+^ < 3.0	33	8	2.86	1.32–6.18	Positive	Positive
Positive	ALT >80	90	47	1.72	1.21–2.44	Positive	Positive

TBil, total bilirubin; ALT, alanine aminotransferase.

### 3.6 Stratified analysis by concomitant medications

For PPIs and aminoglycosides, the aSR estimates were broadly comparable between the co-medication and non-co-medication strata (PPI: 5.00 vs. 5.78; aminoglycosides: 4.58 vs. 5.80), indicating that the main association was not confined to patients receiving these concomitant medications. A larger between-stratum difference was observed for loop diuretics (co-medication: 8.30 vs. non-co-medication: 2.53). In the loop-diuretic non-user stratum, the aSR was 2.53 (95% CI: 0.95–6.74); although the point estimate was above 1, the CI included the null value. Therefore, an association independent of loop-diuretic exposure could not be established from the present data. The estimate was imprecise because only five reverse-sequence events occurred in this stratum, and validation in a larger sample is required (Table 5). Given the small numbers of reverse-sequence events in some strata (npre = 3–5), the stratified results should be interpreted as hypothesis-generating only.

**TABLE 5:** Stratified analysis by concomitant medications.

Medication	Stratum	n_post_	n_pre_	aSR	95% CI
PPI	With	36	5	5.00	1.96–12.74
PPI	Without	22	3	5.78	1.73–19.33
Loop diuretic	With	38	3	8.30	2.56–26.90
Loop diuretic	Without	20	5	2.53	0.95–6.74
Aminoglycoside	With	27	4	4.58	1.60–13.10
Aminoglycoside	Without	31	4	5.80	2.05–16.44

PPI, proton pump inhibitor.

### 3.7 Exploratory dose-response analysis

Among the 532 patients with available weight-based dosing data, 66 (12.4%) developed hypomagnesemia (Mg^2+^ < 0.60 mmol/L) within 90 days of voriconazole initiation. The median weight-based dose was 12.7 mg/kg/day (IQR 8.0–14.6). Across dose tertiles, the 90-day hypomagnesemia rates were 16.2% (low), 10.7% (medium), and 10.2% (high), with no evidence of a dose-response trend (Cochran–Armitage trend test, P = 0.437; high vs. low tertile RR = 0.63, P = 0.146). Weight-based dose did not differ between patients with and without hypomagnesemia (median 11.2 vs. 12.9 mg/kg/day; Mann-Whitney P = 0.336), and the number of voriconazole administrations (a proxy for treatment duration) did not differ between groups (P = 0.254).

## 4 Discussion

Using approximately 10 years of real-world inpatient data from a single children’s hospital and laboratory values rather than prescriptions to define the outcome, this study identified a temporal association between voriconazole initiation and subsequent hypomagnesemia (aSR = 5.76, 95% CI: 2.75–12.06). The association remained statistically significant across all parameter sensitivity analyses and after the exploratory monitoring-intensity correction. The four control analyses agreed with their prespecified expectations, and the direction of the association was generally consistent across the concomitant-medication strata, although the loop-diuretic non-user estimate was imprecise and not statistically significant. Together with the retrospective verification of the available baseline period, these findings support the robustness of the observed temporal association, but they do not by themselves establish causality. PSSA has predominantly been validated using prescription-based outcomes, where both the index drug and the outcome are recorded in dispensing data (Pratt et al., 2015; Wahab et al., 2013). Its application to laboratory-defined outcomes is less established; however, the consistency of the present findings across four prespecified control outcomes supports the internal validity of laboratory-based PSSA, while external validation in other datasets remains desirable.

Building on the existing PSSA methodological literature, this study introduced several targeted refinements tailored to the characteristics of the present dataset. First, retrospective verification of data completeness confirmed that the first voriconazole prescription and first hypomagnesemia event for all patients occurred well after the data extraction start date (2013), with each patient having ≥3 years of blank baseline, naturally satisfying the 365-day washout requirement for both incident users and incident outcomes. This was not a traditional “washout parameter setting” but rather a “factual confirmation” of the incident-user/incident-outcome properties of the dataset—in retrospective PSSA, left-truncation of data may render washout screening ineffective (for patients near the start of the study period, there is effectively insufficient historical data for verification), and the present dataset was fortunately exempt from this limitation. Second, ICD-10 codes have well-defined nosological meanings, and the same set of codes can be precisely reproduced across different researchers and institutional information systems, avoiding both the irreproducibility of study populations caused by different investigator-chosen keyword combinations in fuzzy matching and the false-positive contamination from certain keywords inadvertently matching non-target diagnoses. Third, using the patient rather than the individual hospitalization as the unit of analysis avoided the violation of the PSSA independence assumption that would arise from the repeated counting of the 58.2% of patients with multiple hospitalizations.

The clinical significance of the four thresholds deserves comment. Serum magnesium <0.70 mmol/L marks the lower bound of the normal reference range; values of 0.60–0.69 mmol/L represent mild, usually asymptomatic hypomagnesemia, whereas values <0.50 mmol/L are associated with symptomatic hypomagnesemia (e.g., tetany, seizures, arrhythmias) and warrant urgent replacement in children (Kaplinsky and Alon, 2013). The consistent association across thresholds suggests that even mild hypomagnesemia is temporally linked to voriconazole exposure, while the stability of the aSR estimates indicates that the signal is not driven by the most severely affected patients alone. Conversely, the lack of a monotonic increase in aSR with decreasing threshold argues against a simple severity–response relationship and underscores that the outcome definition—rather than a single cutoff—determines signal detection in laboratory-based PSSA.

The observed temporal distribution—median onset at 28 days, 55.2% of events within 30 days, and the greatest accumulation during weeks 2–4—provides information on when the laboratory abnormality was first detected after voriconazole initiation but does not establish its biological mechanism. Under normal physiological conditions, approximately 2,400 mg of magnesium is filtered by the glomeruli daily, of which more than 95% is reabsorbed along the renal tubule. The distal convoluted tubule mediates approximately 5%–10% of active magnesium reabsorption, partly through the transient receptor potential melastatin 6 (TRPM6) channel on the apical membrane of epithelial cells (de Baaij et al., 2015; de Baaij, 2023). Several drug classes can cause hypomagnesemia by altering magnesium transport: PPIs may affect intestinal magnesium absorption and TRPM6 function (Gommers et al., 2022), whereas calcineurin inhibitors can promote renal magnesium wasting by downregulating renal magnesium transport proteins, including TRPM6 (Nijenhuis et al., 2004). The molecular mechanism underlying the observed voriconazole–hypomagnesemia association remains unknown. The present temporal findings may help define the monitoring period for future studies, but mechanistic investigations are required before involvement of a specific renal or intestinal magnesium transport pathway can be inferred.

The tension between the near-“blindness” of FAERS to voriconazole-associated hypomagnesemia and the strong positive signal observed in this study reflects not the superiority or inferiority of either method but the systematic differences in monitoring windows and signal capture mechanisms. Spontaneous reporting systems rely on clinicians' willingness to report and are inherently biased toward capturing “loud” adverse reactions (severe liver injury, QTc prolongation, Stevens-Johnson syndrome, etc.), while largely losing the ability to monitor “silent” laboratory-abnormality-type adverse reactions. PSSA, by leveraging the existing laboratory records in the HIS without relying on any human reporting action, achieves genuine active surveillance for these “silent ADRs.”

The difference between the ALT-control estimate and the primary hypomagnesemia estimate warrants consideration. Although the ALT aSR of 1.72 was statistically significant, it was lower than the hypomagnesemia aSR of 5.76. ALT elevation is common among pediatric patients with hematologic diseases because of underlying disease, chemotherapy, infections, hemolysis, and hepatobiliary complications, resulting in a high background occurrence both before and after voriconazole initiation. In PSSA, a high frequency of reverse-sequence events can move the sequence ratio toward 1 even when post-exposure events are also increased. Hypomagnesemia was less frequent before voriconazole initiation in this dataset, producing a larger imbalance between the two sequence directions. Accordingly, the difference in aSRs should be interpreted in relation to the different background frequencies and temporal distributions of the two outcomes, rather than as evidence that voriconazole has a stronger causal effect on magnesium than on ALT.

Several limitations of this study should be noted. First, the PSSA design itself cannot control for time-varying confounders (Gault et al., 2017) (e.g., fluctuations in disease severity and chemotherapy intensity), which may lead to overestimation of the aSR. Second, serum magnesium monitoring was not randomly assigned; monitoring frequency after voriconazole initiation may increase owing to heightened clinical vigilance, amplifying the detection rate through monitoring intensity (monitoring bias). This bias could not be completely excluded; however, the median of 20 magnesium measurements per patient among the 364 patients suggests relatively dense monitoring, partially mitigating this concern. Differential laboratory testing could also introduce selection bias, as patients with greater illness severity may have been tested more frequently. In the present cohort, the monitored and unmonitored groups were similar in age (median 8.2 vs. 7.2 years) and ICU admission (2.2% vs. 1.2%), suggesting no major severity-related selection; nevertheless, residual selection effects cannot be excluded. Conservative correction of the main-analysis aSR using the between-group monitoring density ratio (1.73) yielded a value of 3.33 (95% CI: 1.59–6.98), with the signal direction and significance unchanged. Propensity score weighting was not applied because PSSA is a self-controlled design: each patient contributes both pre- and post-exposure observations, and there is no distinct comparison group whose baseline covariates could be balanced by weighting. The ratio-based correction adopted here directly addresses the mechanism of monitoring bias—differential testing intensity between sequence directions—and the sensitivity grid shows that the conclusion is robust to substantial variation in the assumed ratio: the lower confidence bound crosses 1.0 only if the true ratio exceeds 2.75, nearly 1.6-fold the observed value of 1.73. Additional limitations include: single-center data limiting external generalizability; the inability of the HIS to track out-of-hospital medication use and out-of-hospital hypomagnesemia events; the lack of voriconazole therapeutic drug monitoring data precluding concentration–effect analysis; and the possibility of reverse clinical decision-making, whereby clinicians may have avoided or delayed voriconazole in patients with pre-existing hypomagnesemia, which would tend to shift events from the reverse sequence to the post-exposure sequence and could inflate the observed sequence ratio; limited sample sizes in certain stratified-analysis subgroups (loop diuretic non-user stratum n_pre_ = 5; PPI non-user stratum n_pre_ = 3), where the point estimate of aSR is sensitive to single-case changes—each change of one case in n_pre_ can cause the aSR to fluctuate by over 30%, and the 95% CI lower limits were 0.95 (loop diuretic non-user stratum) and 1.73 (PPI non-user stratum), the former already crossing 1.0, indicating limited statistical stability that warrants validation in larger samples.

Implications for clinical practice should be interpreted in light of the observational design. Baseline serum magnesium measurement before voriconazole initiation and periodic monitoring during therapy may be considered, with particular attention during the first 30 days, when more than half of the post-exposure events were detected. Patients receiving concomitant loop diuretics may warrant closer monitoring because the highest point estimate was observed in this stratum, although residual confounding and limited subgroup sizes preclude firm risk-stratification conclusions. When hypomagnesemia is detected, oral or intravenous magnesium supplementation should follow institutional pediatric protocols, and voriconazole discontinuation or dose reduction should be reserved for severe or refractory hypomagnesemia or clinically significant manifestations (e.g., cardiac arrhythmia), in consultation with the infectious diseases team. Voriconazole therapeutic drug monitoring may also be considered when clinically indicated and institutionally available (Abdul-Aziz et al., 2020). In pediatric patients, voriconazole exhibits substantial pharmacokinetic variability (Moriyama et al., 2017), and TDM-guided dosing has been associated with improved treatment outcomes and reduced toxicity across antifungal agents (Resztak et al., 2021; Gazzaniga et al., 2026; Pascual et al., 2008); the present findings on hypomagnesemia add to the case for systematic monitoring of both drug concentrations and electrolytes in this population. More broadly, PSSA may support institutional screening of drug–laboratory-value pairs and complement spontaneous adverse drug reaction reporting, particularly for adverse events primarily detected through laboratory testing. These findings should be regarded as hypothesis-generating evidence supporting a temporal association between voriconazole and hypomagnesemia; confirmation in prospective or multi-center studies is required. Future work should include multi-center validation across different healthcare systems, mechanistic investigations of voriconazole’s effect on renal magnesium handling, and prospective monitoring studies to determine whether routine serum magnesium surveillance improves clinical outcomes in this population.

## Data Availability

The data analyzed in this study is subject to the following licenses/restrictions: The dataset contains confidential patient-level clinical information from the hospital information system of the Children’s Hospital of Chongqing Medical University and is not publicly available under institutional data-protection requirements. De-identified data may be made available from the corresponding author upon reasonable request, subject to institutional authorization. Requests to access these datasets should be directed to LT, linbott@163.com.
