# Supplementary material for: Prescription sequence symmetry analysis of voriconazole-associated hypomagnesemia in pediatric patients with hematologic diseases
Source: Front Pharmacol. 2026 Sep 9;17:1948603. doi: 10.3389/fphar.2026.1948603 (PMC13597875; doi:10.3389/fphar.2026.1948603)
Supplement: Supplementary file 1 [file Supplementaryfile1.docx]

**Supplementary Code: Prescription Sequence Symmetry Analysis of Voriconazole-Associated Hypomagnesemia**

```
#!/usr/bin/env python
"""
Prescription sequence symmetry analysis of voriconazole-associated
hypomagnesemia (revision). Reproduces all results reported in the
manuscript, including sensitivity analyses, Pratt controls,
monitoring-intensity correction, stratified analyses, and the
exploratory dose-response analysis.
"""
import pandas as pd, numpy as np, os, re, json, math
from scipy import stats
from scipy.stats import norm

BASE = os.path.dirname(os.path.abspath(__file__))
INPUT = os.path.join(BASE, '原始数据', '伏立康唑.xlsx')
OUT   = os.path.join(BASE, 'PSSA_Outputs')
os.makedirs(OUT, exist_ok=True)

VERIFY_WIN_START = pd.Timestamp('2013-11-13')
FORMAL_START     = pd.Timestamp('2014-11-13')
DATA_END         = pd.Timestamp('2024-12-04')

# ---- data loading ----
df_drug  = pd.read_excel(INPUT, sheet_name='药物治疗', dtype=str)
df_lab   = pd.read_excel(INPUT, sheet_name='实验室检查_行级数据', dtype=str)
df_diag  = pd.read_excel(INPUT, sheet_name='诊断信息', dtype=str)
df_visit = pd.read_excel(INPUT, sheet_name='就诊信息', dtype=str)
df_pat   = pd.read_excel(INPUT, sheet_name='患者基本信息', dtype=str)
df_adm   = pd.read_excel(INPUT, sheet_name='入院记录', dtype=str)

# ---- population: ICD-10 hematologic diseases ----
heme_icd3 = [f'C{c}' for c in range(81, 97)] + ['D45', 'D46', 'D47', 'D60', 'D61']
mask = df_diag['诊断ICD10编码'].str[:3].isin(heme_icd3)
heme_pts = set(df_diag[mask]['患者编号'].unique())

# ---- voriconazole prescriptions in formal period ----
vrc_pat = r'伏立康唑|威凡|voriconazole'
df_vrc = df_drug[df_drug['药品名称（归一化）'].str.contains(vrc_pat, case=False, na=False)].copy()
df_vrc = df_vrc[df_vrc['患者编号'].isin(heme_pts)]
df_vrc['time'] = pd.to_datetime(df_vrc['医嘱开始时间'], errors='coerce')
df_vrc = df_vrc.dropna(subset=['time'])
df_vrc = df_vrc[df_vrc['time'] >= FORMAL_START]

# ---- laboratory data ----
def load_lab(name, exact=False):
    if exact:
        df = df_lab[df_lab['检验项目名称'] == name].copy()
    else:
        df = df_lab[df_lab['检验项目名称'].str.contains(name, case=False, na=False)].copy()
    df = df[df['患者编号'].isin(heme_pts)]
    df['time'] = pd.to_datetime(df['检验时间'], errors='coerce')
    df['value'] = pd.to_numeric(df['检验定量结果'], errors='coerce')
    return df.dropna(subset=['time', 'value'])

df_mg  = load_lab('镁')
df_mg  = df_mg[df_mg['检验项目名称'] == '镁']  # serum Mg only
df_k   = load_lab('钾离子', exact=True)
df_na  = load_lab('钠离子', exact=True)
df_alt = load_lab('谷丙转氨酶', exact=True)
df_tb  = load_lab('总胆红素', exact=True)

vrc_pts = set(df_vrc['患者编号'].unique())
mg_pts  = set(df_mg['患者编号'].unique())
both = vrc_pts & mg_pts
print(f"monitored patients: {len(both)} ({len(both)/len(vrc_pts)*100:.1f}%)")

# ---- PSSA core ----
def delta_days(t1, t2):
    return (pd.Timestamp(t1) - pd.Timestamp(t2)).total_seconds() / 86400.0

def pssa(df_event, direction, threshold,
         washout=365.0, window=90.0, blackout=7.0, pts_subset=None):
    """Incident users + incident outcomes, time windows, permutation null, aSR."""
    vrc_data = df_vrc if pts_subset is None else df_vrc[df_vrc['患者编号'].isin(pts_subset)]
    fv = vrc_data.groupby('患者编号')['time'].min().reset_index()
    fv.columns = ['pid', 'vrc_date']

    inc = []
    for _, row in fv.iterrows():
        pid, d = row['pid'], row['vrc_date']
        prev = vrc_data[vrc_data['患者编号'] == pid]
        prev_deltas = [delta_days(d, t) for t in prev['time']]
        if not any(0 < dd <= washout for dd in prev_deltas):
            inc.append({'pid': pid, 'vrc_date': d})
    df_inc = pd.DataFrame(inc)
    if len(df_inc) == 0:
        return None

    evt = df_event[df_event['value'] < threshold] if direction == 'lt' else df_event[df_event['value'] > threshold]
    fe = evt.groupby('患者编号')['time'].min().reset_index()
    fe.columns = ['pid', 'evt_date']
    clean = []
    for _, row in fe.iterrows():
        pid, d = row['pid'], row['evt_date']
        prev = evt[(evt['患者编号'] == pid) & (evt['time'] < d)]
        prev_deltas = [delta_days(d, t) for t in prev['time']]
        if not any(0 < dd <= washout for dd in prev_deltas):
            clean.append({'pid': pid, 'evt_date': d})
    df_clean = pd.DataFrame(clean)
    if len(df_clean) == 0:
        return None

    merged = df_inc.merge(df_clean, on='pid', how='inner')
    merged['delta'] = [delta_days(r['evt_date'], r['vrc_date']) for _, r in merged.iterrows()]

    # full pairs (pre-filter) for permutation
    all_vrc_dates = merged['vrc_date'].values
    all_evt_dates = merged['evt_date'].values

    merged = merged[(merged['delta'].abs() > blackout) & (merged['delta'].abs() <= window)]
    n_post = (merged['delta'] > 0).sum()
    n_pre  = (merged['delta'] < 0).sum()
    if n_post + n_pre == 0 or n_pre == 0:
        return None
    cSR = n_post / n_pre

    np.random.seed(42)
    null_ratios = []
    for _ in range(2000):
        shuf = np.random.permutation(all_evt_dates)
        sd = [delta_days(shuf[i], all_vrc_dates[i]) for i in range(len(all_vrc_dates))]
        sd = [d for d in sd if abs(d) > blackout and abs(d) <= window]
        sp = sum(1 for d in sd if d > 0)
        sm = sum(1 for d in sd if d < 0)
        if sm > 0:
            null_ratios.append(sp / sm)
    SR_null = np.mean(null_ratios) if null_ratios else 1.0
    aSR = cSR / SR_null

    se = np.sqrt(1 / n_post + 1 / n_pre)
    ci_low  = np.exp(np.log(aSR) - 1.96 * se)
    ci_high = np.exp(np.log(aSR) + 1.96 * se)

    post_d = merged.loc[merged['delta'] > 0, 'delta'].values
    res = {
        'n_post': int(n_post), 'n_pre': int(n_pre), 'n_total': int(n_post + n_pre),
        'cSR': cSR, 'SR_null': SR_null, 'aSR': aSR,
        'ci_low': ci_low, 'ci_high': ci_high,
        'median_days': float(np.median(post_d)) if len(post_d) else None,
        'pct_30d': float(sum(1 for d in post_d if d <= 30) / len(post_d) * 100) if len(post_d) else None,
        'post_pids': set(merged.loc[merged['delta'] > 0, 'pid']),
        'pre_pids': set(merged.loc[merged['delta'] < 0, 'pid']),
    }
    return res

def fmt(r):
    return f"n_post={r['n_post']} n_pre={r['n_pre']} cSR={r['cSR']:.3f} null={r['SR_null']:.3f} aSR={r['aSR']:.2f} [{r['ci_low']:.2f}, {r['ci_high']:.2f}]"

# ---- main analysis + sensitivity grid ----
print("\nmain + sensitivity")
params = [
    ('Main (Mg<0.60)', 0.6, 365, 90, 7),
    ('Mg<0.55', 0.55, 365, 90, 7),
    ('Mg<0.65', 0.65, 365, 90, 7),
    ('Mg<0.70', 0.70, 365, 90, 7),
    ('Washout 180', 0.6, 180, 90, 7),
    ('Washout 540', 0.6, 540, 90, 7),
    ('Window 30', 0.6, 365, 30, 7),
    ('Window 60', 0.6, 365, 60, 7),
    ('Window 180', 0.6, 365, 180, 7),
    ('Blackout 14', 0.6, 365, 90, 14),
    ('No blackout', 0.6, 365, 90, 0),
]
all_r = {}
main_r = None
for lbl, th, wash, win, blk in params:
    r = pssa(df_mg, 'lt', th, float(wash), float(win), float(blk))
    if r:
        r['label'] = lbl
        all_r[lbl] = r
        print(f"  {lbl:<16s} {fmt(r)}")
    if 'Main' in lbl:
        main_r = r

# ---- Pratt controls ----
print("\nPratt controls")
ctrls = [
    ('Na<130 (true-neg)', df_na, 'lt', 130),
    ('TBil>34 (weak-neg)', df_tb, 'gt', 34),
    ('K<3.0 (weak-pos)', df_k, 'lt', 3.0),
    ('ALT>80 (pos)', df_alt, 'gt', 80),
]
ctrl_r = {}
for name, df_e, dr, th in ctrls:
    r = pssa(df_e, dr, th, 365, 90, 7)
    if r:
        ctrl_r[name] = r
        print(f"  {name:<22s} {fmt(r)}")

# ---- stratified analyses by concomitant medication (±30 d) ----
print("\nstratified by co-medication")
first_vrc_dates = df_vrc.groupby('患者编号')['time'].min().reset_index()
first_vrc_dates.columns = ['pid', 'vrc_date']

drug_defs = [
    ('PPI', '奥美拉唑|泮托拉唑|兰索拉唑|雷贝拉唑|埃索美拉唑|艾司奥美拉唑|艾普拉唑'),
    ('Loop diuretic', '呋塞米|托拉塞米|布美他尼'),
    ('Aminoglycoside', '庆大霉素|阿米卡星|妥布霉素|奈替米星|依替米星'),
]
strata_r = {}
for drug_name, drug_pat in drug_defs:
    df_d = df_drug[df_drug['药品名称（归一化）'].str.contains(drug_pat, case=False, na=False)].copy()
    df_d['time'] = pd.to_datetime(df_d['医嘱开始时间'], errors='coerce')
    df_d = df_d.dropna(subset=['time'])
    pts_with = set()
    for _, row in first_vrc_dates.iterrows():
        pid, vd = row['pid'], row['vrc_date']
        d = df_d[df_d['患者编号'] == pid]
        if len(d) > 0:
            dates = [pd.Timestamp(dd) for dd in d['time'].values]
            if any(abs(delta_days(vd, dd)) <= 30 for dd in dates):
                pts_with.add(pid)
    pts_with &= vrc_pts
    pts_without = vrc_pts - pts_with
    print(f"  {drug_name}: with={len(pts_with)} without={len(pts_without)}")
    for lbl, subset in [('With', pts_with), ('Without', pts_without)]:
        r = pssa(df_mg, 'lt', 0.6, 365, 90, 7, subset)
        if r:
            strata_r[f'{drug_name}_{lbl}'] = r
            print(f"    {lbl:<8s} {fmt(r)}")

# ---- monitoring-intensity correction (counts within 90 d after VRC) ----
print("\nmonitoring-intensity correction")
post_pids = main_r['post_pids']
pre_pids  = main_r['pre_pids']

def counts_in_90d(pid_set):
    cnts = {}
    for pid in pid_set:
        vd = first_vrc_dates.loc[first_vrc_dates['pid'] == pid, 'vrc_date'].iloc[0]
        sub = df_mg[(df_mg['患者编号'] == pid) &
                    (df_mg['time'] >= vd) &
                    (df_mg['time'] <= vd + pd.Timedelta(days=90))]
        cnts[pid] = len(sub)
    return cnts

pc = counts_in_90d(post_pids)
qc = counts_in_90d(pre_pids)
post_mean = np.mean(list(pc.values())); post_med = np.median(list(pc.values()))
pre_mean  = np.mean(list(qc.values()));  pre_med  = np.median(list(qc.values()))
ratio = post_mean / pre_mean
print(f"  post: mean {post_mean:.1f} (median {post_med:.1f})  pre: mean {pre_mean:.1f} (median {pre_med:.1f})")
print(f"  ratio: {ratio:.2f}")
aSR_corr = main_r['aSR'] / ratio
ci_corr = (main_r['ci_low'] / ratio, main_r['ci_high'] / ratio)
print(f"  corrected: aSR={aSR_corr:.2f} [{ci_corr[0]:.2f}, {ci_corr[1]:.2f}]")

grid = {}
for rr in [1.35, 1.73, 2.0, 2.5, 2.75, 3.0]:
    a = main_r['aSR'] / rr
    lo = main_r['ci_low'] / rr
    grid[rr] = {'aSR': round(a, 2), 'ci_low': round(lo, 2)}
    print(f"    ratio={rr} -> aSR={a:.2f} [{lo:.2f}, ...]")
with open(os.path.join(OUT, 'monitoring_ratio_sensitivity.json'), 'w') as f:
    json.dump(grid, f, indent=2)

# ---- dose-response analysis ----
print("\ndose-response")
def parse_weight(s):
    if pd.isna(s): return np.nan
    m = re.search(r'([\d.]+)\s*(kg|Kg|KG|千克)?', str(s))
    return float(m.group(1)) if m else np.nan

df_adm['weight_kg'] = df_adm['入院记录体重'].apply(parse_weight)
df_adm['weight_kg'] = df_adm['weight_kg'].where(df_adm['weight_kg'].between(3, 150))
df_adm['visit'] = df_adm['就诊编号（挂号编号，住院就诊编号）'].astype(str)
# one record per admission (deduplicate duplicate chart rows per visit)
adm_w = df_adm.dropna(subset=['weight_kg']).groupby('visit')['weight_kg'].max().reset_index()

def freq_per_day(f):
    if pd.isna(f): return 1.0
    f = str(f).strip()
    m = re.match(r'(\d+)/(\d+)小时', f)
    if m: return int(m.group(1)) * 24 / int(m.group(2))
    m = re.match(r'(\d+)/日', f)
    if m: return float(m.group(1))
    if f in ('1/早', '1/晚'): return 1.0
    if f.upper() == 'Q2H': return 12.0
    return 1.0

def dose_mg(val, unit):
    try: v = float(val)
    except: return np.nan
    if unit in ('mg', '毫克'): return v
    if unit == 'g': return v * 1000
    if unit in ('粒', '片'): return v * 200
    return np.nan

vrc_all = df_vrc.copy()
vrc_all['就诊编号'] = vrc_all['就诊编号'].astype(str)
vrc_all['end'] = pd.to_datetime(vrc_all['医嘱结束时间'], errors='coerce')
vrc_all['freq'] = vrc_all['用药频次'].apply(freq_per_day)
vrc_all['dose_mg'] = [dose_mg(d, u) for d, u in zip(vrc_all['药物单次剂量'], vrc_all['药物剂量单位'])]
vrc_all['daily_mg'] = vrc_all['dose_mg'] * vrc_all['freq']

fv = vrc_all.sort_values('time').drop_duplicates('患者编号')[['患者编号', 'time', '就诊编号']]
fv.columns = ['患者编号', 'first_vrc_time', 'first_visit_id']
course = vrc_all.merge(fv[['患者编号', 'first_vrc_time']], on='患者编号', how='left')
course = course[(course['time'] >= course['first_vrc_time']) &
                (course['time'] <= course['first_vrc_time'] + pd.Timedelta(days=14))]
per = course.groupby('患者编号').agg(max_daily_mg=('daily_mg', 'max'), n_orders=('daily_mg', 'size')).reset_index()
per = per.merge(fv[['患者编号', 'first_visit_id', 'first_vrc_time']], on='患者编号', how='left')
per = per.merge(adm_w, left_on='first_visit_id', right_on='visit', how='left')
per['mg_per_kg_per_day'] = per['max_daily_mg'] / per['weight_kg']

fe = df_mg[df_mg['value'] < 0.6].sort_values('time').drop_duplicates('患者编号')[['患者编号', 'time']]
fe.columns = ['患者编号', 'first_hypo_time']
per = per.merge(fe, on='患者编号', how='left')
per['hypo_within_90d'] = ((per['first_hypo_time'] - per['first_vrc_time']).dt.total_seconds().div(86400).between(0, 90)).astype(int)

with_wt = per.dropna(subset=['mg_per_kg_per_day']).copy()
print(f"  with dose+weight: {len(with_wt)}; hypo within 90d: {with_wt['hypo_within_90d'].sum()} ({with_wt['hypo_within_90d'].mean()*100:.1f}%)")
print(f"  median dose: {with_wt['mg_per_kg_per_day'].median():.1f} (IQR {with_wt['mg_per_kg_per_day'].quantile(.25):.1f}-{with_wt['mg_per_kg_per_day'].quantile(.75):.1f})")

with_wt['dose_tertile'] = pd.qcut(with_wt['mg_per_kg_per_day'], 3, labels=['Low', 'Medium', 'High'], duplicates='drop')
tab = with_wt.groupby('dose_tertile', observed=True).agg(n=('hypo_within_90d', 'size'), hypo=('hypo_within_90d', 'sum')).reset_index()
tab['rate_%'] = (tab['hypo'] / tab['n'] * 100).round(1)
print(tab.to_string(index=False))

# Cochran-Armitage trend test
n = tab['n'].values; x = tab['hypo'].values
N = n.sum(); pbar = x.sum() / N
scores = np.arange(1, len(n) + 1)
T = (scores * (x - n * pbar)).sum()
var_T = pbar * (1 - pbar) * ((scores**2 * n * (N - n) / N).sum())
Z = T / np.sqrt(var_T)
p_ca = 2 * (1 - norm.cdf(abs(Z)))
chi2_trend = T**2 / var_T
p_ca2 = stats.chi2.sf(chi2_trend, 1)
lo, hi = tab.iloc[0], tab.iloc[-1]
rr = (hi['hypo'] / hi['n']) / (lo['hypo'] / lo['n'])
chi2, p_rr = stats.chi2_contingency([[lo['hypo'], lo['n'] - lo['hypo']], [hi['hypo'], hi['n'] - hi['hypo']]])[:2]
u, p_mw = stats.mannwhitneyu(with_wt.loc[with_wt['hypo_within_90d'] == 1, 'mg_per_kg_per_day'],
                             with_wt.loc[with_wt['hypo_within_90d'] == 0, 'mg_per_kg_per_day'], alternative='two-sided')
u2, p_dur = stats.mannwhitneyu(with_wt.loc[with_wt['hypo_within_90d'] == 1, 'n_orders'],
                               with_wt.loc[with_wt['hypo_within_90d'] == 0, 'n_orders'], alternative='two-sided')
print(f"  CA: Z={Z:.3f}, p={p_ca:.3f} (chi2(1) p={p_ca2:.3f})")
print(f"  High vs Low: RR={rr:.2f}, p={p_rr:.3f}")
print(f"  MW: p={p_mw:.3f}")
print(f"  n_orders: p={p_dur:.3f}")
with_wt.to_csv(os.path.join(OUT, 'dose_response_patient_data_dedup.csv'), index=False, encoding='utf-8-sig')

# ---- baseline characteristics ----
print("\nbaseline")
print(f"  VRC patients (formal): {len(vrc_pts)}")
print(f"  monitored: {len(both)} ({len(both)/len(vrc_pts)*100:.1f}%)")
pat_both = df_pat[df_pat['患者编号'].isin(both)]
df_case = pd.read_excel(INPUT, sheet_name='病案基本信息', dtype=str)
case_sex = df_case.groupby('患者编号')['性别名称'].first()
def fill_sex(pid):
    s = df_pat.loc[df_pat['患者编号'] == pid, '性别']
    if len(s) and s.iloc[0] not in ('未知的性别',) and not pd.isna(s.iloc[0]):
        return s.iloc[0]
    if pid in case_sex.index:
        return case_sex[pid]
    return '未知的性别'
pat_both['性别'] = pat_both['患者编号'].apply(fill_sex)
print(f"  sex (monitored): {dict(pat_both['性别'].value_counts())}")

age_pat = pd.to_numeric(pat_both['年龄（天）'], errors='coerce') / 365.25
print(f"  age-patient table: median {age_pat.median():.1f} (IQR {age_pat.quantile(.25):.1f}-{age_pat.quantile(.75):.1f}, range {age_pat.min():.1f}-{age_pat.max():.1f})")
vrc_first = df_vrc.sort_values('time').drop_duplicates('患者编号')
vrc_first = vrc_first[vrc_first['患者编号'].isin(both)]
age_merged = vrc_first[['患者编号', '就诊编号']].merge(
    df_visit[['患者编号', '就诊编号', '年龄换算（天）']], on=['患者编号', '就诊编号'], how='left')
ages_v = pd.to_numeric(age_merged['年龄换算（天）'], errors='coerce').dropna() / 365.25
print(f"  age-first VRC visit: median {ages_v.median():.1f} (IQR {ages_v.quantile(.25):.1f}-{ages_v.quantile(.75):.1f}, range {ages_v.min():.1f}-{ages_v.max():.1f})")
mg_counts = df_mg[df_mg['患者编号'].isin(both)].groupby('患者编号').size()
mg_vals = df_mg[df_mg['患者编号'].isin(both)]['value']
print(f"  Mg measurements: {mg_counts.sum()}, median/patient {mg_counts.median():.0f} (IQR {mg_counts.quantile(.25):.0f}-{mg_counts.quantile(.75):.0f}), median value {mg_vals.median():.2f}")

vis = df_visit[df_visit['患者编号'].isin(vrc_pts)]
vc = vis.groupby('患者编号').size()
print(f"  visits: {vc.sum()}, >=2 hospitalizations: {(vc>=2).sum()} ({(vc>=2).sum()/len(vrc_pts)*100:.1f}%)")
vis_icu = vis.copy()
vis_icu['is_icu'] = vis_icu['就诊科室'].astype(str).str.contains('重症|ICU|PICU|NICU', na=False)
icu_n = vis_icu[vis_icu['患者编号'].isin(both)].groupby('患者编号')['is_icu'].any().sum()
print(f"  ICU (monitored): {icu_n} ({icu_n/len(both)*100:.1f}%)")
renal_codes = ['N0','N1','N2','N3','N4','N5','N7','N8','I12','I13']
renal_pts = set(df_diag[df_diag['诊断ICD10编码'].str[:3].isin(renal_codes)]['患者编号'])
print(f"  renal disease (monitored): {len(both & renal_pts)}; (unmonitored): {len((vrc_pts-mg_pts) & renal_pts)}")
for drug_name, drug_pat in drug_defs:
    df_d = df_drug[df_drug['药品名称（归一化）'].str.contains(drug_pat, case=False, na=False)].copy()
    df_d['time'] = pd.to_datetime(df_d['医嘱开始时间'], errors='coerce')
    df_d = df_d.dropna(subset=['time'])
    cnt = 0
    for _, row in first_vrc_dates.iterrows():
        pid, vd = row['pid'], row['vrc_date']
        if pid not in both: continue
        d = df_d[df_d['患者编号'] == pid]
        if len(d) > 0:
            dates = [pd.Timestamp(dd) for dd in d['time'].values]
            if any(abs(delta_days(vd, dd)) <= 30 for dd in dates):
                cnt += 1
    print(f"  co-med (monitored, ±30d) {drug_name}: {cnt} ({cnt/len(both)*100:.1f}%)")

# ---- monitored vs unmonitored ----
print("\nmonitored vs unmonitored")
unmon = vrc_pts - mg_pts
age_pat_all = pd.to_numeric(df_pat['年龄（天）'], errors='coerce') / 365.25
df_pat2 = df_pat.copy(); df_pat2['age_y'] = age_pat_all
print(f"  age: monitored {df_pat2[df_pat2['患者编号'].isin(both)]['age_y'].median():.1f} vs unmonitored {df_pat2[df_pat2['患者编号'].isin(unmon)]['age_y'].median():.1f}")
icu_m = vis_icu[vis_icu['患者编号'].isin(both)].groupby('患者编号')['is_icu'].any().mean()
icu_u = vis_icu[vis_icu['患者编号'].isin(unmon)].groupby('患者编号')['is_icu'].any().mean()
print(f"  ICU: monitored {icu_m*100:.1f}% vs unmonitored {icu_u*100:.1f}%")
sex_m = df_pat2[df_pat2['患者编号'].isin(both)].assign(sex_f=lambda d: d['患者编号'].map(fill_sex))['sex_f'].value_counts()
sex_u = df_pat2[df_pat2['患者编号'].isin(unmon)].assign(sex_f=lambda d: d['患者编号'].map(fill_sex))['sex_f'].value_counts()
print(f"  sex: monitored {dict(sex_m)} unmonitored {dict(sex_u)}")

# ---- annual first-time users ----
print("\nannual first-time voriconazole users (formal period)")
annual = df_vrc.sort_values('time').drop_duplicates('患者编号').groupby(df_vrc.sort_values('time').drop_duplicates('患者编号')['time'].dt.year).size()
print(annual.to_string())

# ---- ICD-10 code table ----
diag = df_diag[df_diag['患者编号'].isin(heme_pts)]
codes = diag[diag['诊断ICD10编码'].str[:3].isin(heme_icd3)]
code_counts = codes.groupby('诊断ICD10编码').agg(n_patients=('患者编号', 'nunique')).reset_index().sort_values('n_patients', ascending=False)
print(f"\nICD-10 codes: {len(code_counts)}")

# ---- summary JSON ----
summary = {
    'main': {k: (v if not isinstance(v, set) else len(v)) for k, v in main_r.items() if k not in ('post_pids','pre_pids')},
    'sensitivity': {k: {kk: (vv if not isinstance(vv, set) else len(vv)) for kk, vv in v.items() if kk not in ('post_pids','pre_pids')} for k, v in all_r.items()},
    'controls': {k: {kk: (vv if not isinstance(vv, set) else len(vv)) for kk, vv in v.items() if kk not in ('post_pids','pre_pids')} for k, v in ctrl_r.items()},
    'strata': {k: {kk: (vv if not isinstance(vv, set) else len(vv)) for kk, vv in v.items() if kk not in ('post_pids','pre_pids')} for k, v in strata_r.items()},
    'monitoring': {'post_mean': post_mean, 'post_med': post_med, 'pre_mean': pre_mean,
                   'pre_med': pre_med, 'ratio': ratio,
                   'aSR_corrected': aSR_corr, 'ci_low_corr': ci_corr[0], 'ci_high_corr': ci_corr[1]},
    'dose_response': {
        'n': int(len(with_wt)), 'n_hypo': int(with_wt['hypo_within_90d'].sum()),
        'hypo_pct': round(with_wt['hypo_within_90d'].mean() * 100, 1),
        'median_dose': float(with_wt['mg_per_kg_per_day'].median()),
        'iqr_dose': [float(with_wt['mg_per_kg_per_day'].quantile(.25)), float(with_wt['mg_per_kg_per_day'].quantile(.75))],
        'tertile_rates': tab['rate_%'].tolist(),
        'ca_z': float(Z), 'ca_p': float(p_ca),
        'rr': float(rr), 'rr_p': float(p_rr),
        'mw_p': float(p_mw), 'dur_p': float(p_dur),
    },
}
with open(os.path.join(OUT, 'pssa_v50_summary.json'), 'w', encoding='utf-8') as f:
    json.dump(summary, f, indent=2, default=str)
print(f"\nresults exported to {OUT}")
```
