# Supplementary material for: Prescription sequence symmetry analysis of voriconazole-associated hypomagnesemia in pediatric patients with hematologic diseases
Source: Front Pharmacol. 2026 Sep 9;17:1948603. doi: 10.3389/fphar.2026.1948603 (PMC13597875; doi:10.3389/fphar.2026.1948603)
Supplement: Supplementary file 2 [file Supplementaryfile2.docx]

**Supplementary Table S1. ICD-10 codes used to identify patients with hematologic diseases.**

Complete list of the 160 International Classification of Diseases, 10th Revision (ICD-10) codes used to define the study population (C81–C96, D45–D47, and D60–D61). The number of patients carrying each code is shown; patients may carry more than one code.

| **ICD-10 code** | **Patients, n** | **ICD-10 code** | **Patients, n** | **ICD-10 code** | **Patients, n** |
| --- | --- | --- | --- | --- | --- |
| D61.101 | 486 | C85.721 | 3 | C92.100x007 | 1 |
| C91.000 | 209 | C85.100y001 | 3 | C92.200x011 | 1 |
| C95.000 | 157 | C81.100x001 | 3 | C92.400x001 | 1 |
| C91.000y002 | 88 | C83.200y001 | 3 | C92.400 | 1 |
| C94.700x004 | 64 | C91.003y002 | 3 | C92.000x002 | 1 |
| D61.900 | 60 | D47.900x001y002 | 3 | C91.200 | 1 |
| C92.000x004 | 56 | D61.007 | 3 | C92.000x003 | 1 |
| C95.900 | 50 | D46.700x003 | 3 | C92.000x016 | 1 |
| C92.000y002 | 49 | C94.001y001 | 3 | C90.000 | 1 |
| C91.002y002 | 43 | C94.700x003 | 3 | C91.002y003 | 1 |
| C91.000y001 | 38 | D61.902 | 3 | C91.003y004 | 1 |
| C91.001y002 | 33 | D47.900x001 | 2 | C90.001 | 1 |
| C91.002 | 31 | D46.700x006 | 2 | C91.000x021 | 1 |
| C91.002y001 | 25 | D61.800 | 2 | C88.900x001 | 1 |
| D61.903y001 | 21 | C94.700x014 | 2 | C85.900y002 | 1 |
| C85.900x006 | 18 | C96.900y001 | 2 | C85.900x040 | 1 |
| C91.001 | 18 | C93.000x022 | 2 | C91.005 | 1 |
| D46.900 | 18 | C95.900y003 | 2 | C92.000x014 | 1 |
| D61.905 | 15 | C96.702 | 2 | C92.001 | 1 |
| C91.006 | 13 | C91.901 | 2 | C92.003 | 1 |
| C92.000 | 11 | C91.001y003 | 2 | C92.901 | 1 |
| D61.903 | 11 | C91.002y005 | 2 | C92.704 | 1 |
| C91.001y004 | 10 | C91.003 | 2 | C92.702 | 1 |
| C95.900y002 | 9 | C91.000x009 | 2 | C92.500x021 | 1 |
| C91.000x006 | 9 | C90.205 | 2 | C92.500x001 | 1 |
| C91.001y001 | 9 | C92.000x019 | 2 | C92.400x021 | 1 |
| D61.903y003 | 8 | C83.306 | 2 | C95.000x018 | 1 |
| C83.400y002 | 8 | C84.500 | 2 | C95.700x001 | 1 |
| C91.002y004 | 8 | C85.000 | 2 | C93.000 | 1 |
| C91.000x013 | 8 | C84.506 | 2 | C94.000 | 1 |
| D61.900x001 | 8 | D61.802 | 2 | C93.000x021 | 1 |
| C83.400y001 | 7 | C92.300 | 2 | C93.900 | 1 |
| D61.909 | 7 | C91.303 | 2 | C95.000x015 | 1 |
| D46.902 | 7 | C94.700 | 2 | C94.000x011 | 1 |
| C83.700y001 | 6 | C92.100x001 | 2 | C95.000x016 | 1 |
| C95.000x006 | 6 | C83.200y002 | 1 | C95.000x116 | 1 |
| C95.900y007 | 5 | C81.100y001 | 1 | C96.705 | 1 |
| C91.001_M98210/3 | 5 | C81.200x002 | 1 | C96.703 | 1 |
| C91.000x012 | 5 | C83.300x006 | 1 | C96.700x002 | 1 |
| D61.801 | 5 | C83.305 | 1 | C96.701 | 1 |
| D47.900x002 | 5 | C83.701 | 1 | C96.101 | 1 |
| C95.901 | 5 | C84.400 | 1 | D46.000x003 | 1 |
| C81.900x001 | 5 | C85.100x031 | 1 | D46.900x002 | 1 |
| C95.900y006 | 4 | C85.100x001y001 | 1 | D46.901 | 1 |
| D60.900x001 | 4 | C85.709 | 1 | D47.100x003 | 1 |
| C91.003y003 | 4 | C84.50001 | 1 | D47.100x006 | 1 |
| C92.500 | 4 | C84.500y002 | 1 | D47.700x007 | 1 |
| C92.000y001 | 4 | C84.500x004 | 1 | D47.703 | 1 |
| C85.900 | 4 | C85.000y003 | 1 | D46.700x008 | 1 |
| C94.000x021 | 3 | C85.100 | 1 | D61.001 | 1 |
| C91.304 | 3 | C83.300 | 1 | D61.003 | 1 |
| C91.001y005 | 3 | C92.004 | 1 | D61.906y001 | 1 |
| C85.900x010 | 3 | C92.200x001 | 1 |  |  |
| C81.900x004 | 3 | C92.100x008 | 1 |  |  |

Abbreviations: ICD-10, International Classification of Diseases, 10th Revision. Patients may carry more than one code; therefore, the sum of patients across codes exceeds the total number of patients (n = 536).
